# Sex Differences in Electrical Activity During Sleep: A Synthesis of Brain Wave Data Across Human Life Stages

**DOI:** 10.1002/alz.095592

**Published:** 2025-01-09

**Authors:** Christy Chi Kiu Lee, Rhea Chapman, Jessica Babineau, Thaisa Tylinski Sant’Ana, Tatyana Mollayeva

**Affiliations:** ^1^ KITE Research Institute Toronto Rehabilitation Institute University Health Network, Toronto, ON Canada; ^2^ Library and Information Services, University Health Network, Toronto, Ontario, Canada, Toronto, ON Canada; ^3^ The Institute for Education Research, University Health Network, Toronto, ON Canada; ^4^ Dalla Lana School of Public Heath University of Toronto, Toronto, ON Canada; ^5^ Global Brain Health Institute, Institute of Neuroscience, Trinity College, Dublin, ON Canada; ^6^ KITE Research Institute University Health Network, Toronto, ON Canada; ^7^ Rehabilitation Sciences Institute Temetry Faculty of Medicine University of Toronto, Toronto, ON Canada

## Abstract

**Background:**

With the explosion of techniques for recording brain activity, including the analysis of electrical signals generated during sleep, our understanding of neural dynamics has expanded significantly. Yet, uncertainty exists regarding whether there are sex differences in brain activity during sleep across the human lifespan. We aimed to address the gap by analyzing published evidence on the topic.

**Method:**

We developed a search strategy to capture English language publications on the topic. Medline ALL, Embase Classic + Embase, APA PsycInfo, Scopus, and Proquest Dissertations and Theses Global were searched in November 2021. We included original studies that reported on sex differences in electrical activity in sleep. No restrictions were applied to study methodology or quality. We analyzed results by frequency bands: delta (0.5‐4 Hz), theta (4‐7 Hz), alpha (8‐12 Hz), sigma (12‐16 Hz), beta (13‐13 Hz), and gamma (30‐80+ Hz) waves. We organized results by age bins of human lifespan stages.

**Result:**

Two independent researchers screened 2,783 citations. We included 29 studies that met our inclusion and exclusion criteria. The most frequently studied sleep activities were delta and theta, reported in 11 studies (38%) each. Eight studies (28%) reported on sex difference in alpha and sigma activity, four (14%) on beta activity, and two (7%) on gamma activity. Within these studies, recording techniques and frequency bands assigned to electrical brain activity varied. The most frequently studied age groups were teenagers (18 studies (62%) focusing on delta and theta waves) and middle to late age adults (10 studies (34%) focusing on delta and alpha waves). No studies reported on sex differences in infancy and early childhood. Age‐dependent results, although inconsistent, supported sex difference around the timing of brain maturation and sex variability during ageing.

**Conclusion:**

The clinical implications of our findings are challenging to examine since most evidence had no direct links to outcomes. Nonetheless, the findings offer insight on the development of future research on sex difference in electrical activity in sleep that may be used as clinical trial outcomes, considering that cognitive, behavioral, and emotional processes are reported to differ between the sexes as they age. PROSPERO: CRD42022327644

